# Identifying functional roles and pathways of shared mutations in canine solid tumors by whole-genome sequencing

**DOI:** 10.1371/journal.pone.0307792

**Published:** 2025-05-30

**Authors:** YeSeul Jeon, Hyeona Bae, Seung-Wan Woo, Jaemin Kim, DoHyeon Yu

**Affiliations:** 1 College of Veterinary Medicine, Gyeongsang National University, Jinju, Republic of Korea; 2 Division of Applied Life Science, Gyeongsang National University, Jinju, Republic of Korea; 3 Institute of Agriculture and Life Sciences, Gyeongsang National University, Jinju, Republic of Korea; University of Vermont College of Medicine, UNITED STATES OF AMERICA

## Abstract

Identifying genetic mutations contributing to solid tumors by altering the biological pathways related to tumor formation and development is essential for the development of targeted therapies. This study aimed to identify commonly mutated genes and altered pathways in canine solid tumors. Four dogs with different types of naturally occurring neoplasias (urothelial carcinoma, adenocarcinoma, rhabdomyosarcoma, and chondrosarcoma) were randomly selected and classified into carcinoma and sarcoma groups based on histopathological findings. Tumor tissues were analyzed using whole-genome sequencing, and significant variants shared within each tumor group were identified. Gene set enrichment analyses were conducted to compare the biological and functional pathways altered by the mutations in each carcinoma and sarcoma group. Forty-three and fifty-eight genes were identified in the carcinoma and sarcoma groups, respectively. Distinctions between the two tumor groups were noted for mutations related to tumor metastatic function. Mutations were identified in genes encoding cell adhesion molecules in the carcinoma group, whereas significant variations in extracellular matrix-related molecules were evident in the sarcoma group. This study revealed mutations and modified pathways associated with immune and tumor metastatic functions in canine carcinoma and sarcoma, indicating their significant relevance to the development and progression of each tumor group. Additionally, the distinctions indicated that different therapeutic approaches were required for each tumor group.

## Introduction

Tumors are diseases caused by genetic mutations that are fundamental to the hallmarks of cancer and lead to their clinical behavior [1]. Each tumor comprises a myriad of diverse mutations (intra-tumor heterogeneity), and individual patients with tumors can harbor unique genetic alterations and characteristics (inter-patient heterogeneity) [2]. To better understand tumor development, it is necessary to identify gene mutations that promote tumor growth and the biological pathways involved in this process [2].

Oncology in human medicine has undergone a paradigm shift to therapies targeting mutations and pathway dysregulation unique to cancers in individual patients using methods such as whole-genome sequencing (WGS), whole-exome sequencing, or targeted sequencing [3]. Targeted chemotherapy selectively targets cancer cells through tumor genomic profiling, minimizing damage to healthy tissues and improving patients’ quality of life [4]. Examples of targeted therapies currently approved and used include small-molecule inhibitors targeting the epidermal growth factor receptor gene and programmed cell death protein 1/programmed cell death ligand 1 inhibitors in tumors with high microsatellite instability [5–7].

However, such research is lacking in veterinary medicine, and the landscape of actionable tumor mutations in canine cancers is not fully understood [8]. Although research has been conducted on molecular targets and their expression in cancers related to companion animals, there are few approved targeted therapies for solid tumors in veterinary medicine. In addition, conducting personalized research for each tumor type is difficult in veterinary medicine because of economic factors, including lack of samples and limited research funding [4]. Hence, this study categorized tumors as carcinomas or sarcomas rather than focusing on specific tumor types.

Carcinomas and sarcomas were selected among solid tumor types because they have distinct etiological origins and exhibit significant differences in their metastatic processes and clinical characteristics [9,10]. These differences are also evident from the perspective of genetic mutations. As in the case of carcinoma, a common set of driver mutations exists in each cancer type, and several recurrent major driver mutations have been investigated [11]. However, the overall burden of somatic mutations in sarcomas is lower than in carcinomas [12], and only a limited number of recurrent mutations occur across various sarcoma subtypes [13,14]. Given the distinctions between the two tumor groups, the mutation patterns and pathways involved in tumor formation were also expected to differ.

This study used WGS analysis to identify single nucleotide variants (SNVs) in carcinoma and sarcoma groups. Additionally, we aimed to elucidate the common mechanisms of the altered pathways within tumors by identifying the biological and functional pathways affected by these SNVs that collectively impact tumor development. These findings provide a foundation for applying therapies used in human medicine that target these pathways in veterinary medicine and may even contribute to developing new therapies.

## Materials and methods

### Study design and case inclusion for WGS

#### Case selection for tumor groups.

From 2021 to 2023, the medical records of dogs diagnosed with solid tumors belonging to the carcinoma or sarcoma types were reviewed at the Veterinary Teaching Hospital, Gyeongsang National University (GNU), to acquire signalment, clinicopathologic, and histopathological diagnosis data. Clinicopathological evaluation and diagnostic imaging, including computed tomography (CT), were conducted at the initial presentation of all four dogs enrolled in the study. Additionally, histopathological data for tumor diagnosis (obtained from IDEXX Laboratories, Westbrook, ME, USA) were collected. The tissue from each tumor was collected for WGS analysis. None of the dogs received chemotherapy or radiation therapy before tissue resection. Immediately after tumor resection, at least 1 g of tumor tissue was placed in an Eppendorf (EP) tube and stored in a deep freezer at -80˚C until analysis. The study protocol was conducted with the approval of the Institutional Animal Care and Committees (IACUC) GNU-231109-D0213 of GNU. The characteristics of the dogs included in this study are summarized in Table 1, and their laboratory data are summarized in Table 2. Detailed histopathological findings and CT results for each dog are included in the supporting information S1A and S1B Fig.

**Table 1:** Signalment of four dogs diagnosed with solid tumors.

Dog	Age	Sex	Breeds	B.W.	Diagnosis	Diagnostic methods	Survival time
**1**	12 years	F	Dachshund	5.1 kg	Urothelial carcinoma	Post-mortem biopsy	319 days
**2**	11 years	NM	Spitz	11.7 kg	Intestinal adenocarcinoma	Surgical biopsy	> 264 days
**3**	5 years	M	Poodle	4.5 kg	Nasal chondrosarcoma	Tru-cut biopsy	24 days
**4**	8 years	M	Borzoi	39.2 kg	Rhabdomyosarcoma	Tru-cut biopsy	Lost to follow-up

F, female; NM, neutered male; M, male; B.W., body weight

**Table 2:** Clinicopathologic analysis of four dogs diagnosed with solid tumors.

CBC	Dog 1	Dog 2	Dog 3	Dog 4	Reference intervals	Serum biochemistry profiles	Dog 1	Dog 2	Dog 3	Dog 4	Reference intervals
**RBC**	6.83	5.98	6.66	6.17	5.65–8.87 (× 10^9^/L)	**Glucose**	122	122	100	113	74–143 (mg/dL)
**HCT**	41.3	26.1	45.0	42.5	37.3–61.7 (%)	**Creatinine**	0.5	0.9	0.4	0.7	0.5–1.8 (mg/dL)
**Hemoglobin**	14.2	7.8	15.2	14.6	13.1–20.5 (g/dL)	**Urea nitrogen**	16	5	6	17	7–27 (mg/dL)
**MCV**	60.5	43.6	67.6	68.9	61.6–73.5 (fL)	**Phosphorus**	4.4	4.0	4.6	3.9	2.5–6.8 (mg/dL)
**MCHC**	34.4	29.9	33.8	34.4	32–37.9 (g/dL)	**Calcium**	9.4	9.1	9.1	10	7.9–12 (mg/dL)
**White blood cells**	42.65	15.85	9.59	7.00	5.05–16.76 (× 10^9^/L)	**Total protein**	7.3	5.7	7.3	7.0	5.2–8.2 (mg/dL)
**Neutrophils**	35.99	12.87	5.51	5.89	2.95–11.64 (× 10^9^/L)	**Albumin**	3.0	2.4	3.0	3.3	2.2–3.9 (g/dL)
**Lymphocytes**	3.69	1.68	2.52	0.66	1.05–5.10 (× 10^9^/L)	**Globulin**	4.3	3.3	4.3	3.6	2.5–4.5 (g/dL)
**Monocytes**	2.88	0.60	1.19	0.32	0.16–1.12 (× 10^9^/L)	**ALT**	54	25	54	75	10–125 (U/L)
**Eosinophils**	0.06	0.64	0.36	0.05	0.06–1.23 (× 10^9^/L)	**ALP**	268	51	268	184	23–212 (U/L)
**Basophils**	0.03	0.06	0.01	0.08	0.00–0.10 (× 10^9^/L)	**GGT**	1	0	7	1	0–11 (U/L)
**Platelet count**	342	511	310	364	148–484 (× 10^9^/L)	**T-bil**	0.3	0.1	0.1	0.7	0–0.9 (mg/dL)
						**Cholesterol**	274	167	176	120	110–320 (mg/dL)
						**Amylase**	590	743	381	479	500–1,500 (U/L)
						**Lipase**	376	505	195	873	200–1,800 (U/L)

CBC, complete blood cell count; RBC, red blood cells; HCT, hematocrit; MCV, mean red cell volume; MCHC, mean cell hemoglobin concentration; WBC, white blood cells; ALT, alanine aminotransferase; ALP, alkaline phosphatase; GGT, gamma-glutamyl transferase; T-bil, total bilirubin

The carcinoma group included dogs 1 and 2. Dog 1 was a 12-year-old female Dachshund breed diagnosed with urothelial carcinoma within the bladder via post-mortem histopathological examination. Despite definitive radiation therapy (40Gy, 20 fractions), the dog survived for 319 days. Dog 2 was an 11-year-old neutered male Spitz breed and was diagnosed with intestinal adenocarcinoma via histopathological examination following surgical excision at the time of diagnosis. After carboplatin-based chemotherapy, the dog survived for 264 days until the end of the study period. Paraneoplastic syndromes of epithelial tumors, including hypercalcemia and hypoglycemia, were not identified in dogs 1 and 2.

The sarcoma group included dogs 3 and 4. Dog 3 was a 5-year-old male poodle, and a diagnosis of nasal chondrosarcoma was established via histopathological examination following a Tru-cut biopsy at the time of diagnosis. Dog 3 was euthanized at the owner’s request, and the ST duration was 24 days. Dog 4 was an 8-year-old male, Borzoi breed; a diagnosis of rhabdomyosarcoma was established via histopathological examination and immunohistochemistry following a Tru-cut biopsy at the time of diagnosis. Dog 4 was lost to follow-up, and ST could not be defined.

#### Case selection for control group.

Signalment data used in the control group was gathered from the Sequence Read Archive (SRA; http://www.ncbi.nlm.nih.gov/sra; n = 52 unique individuals), contributed by collaborators (n = 128) or generated by the NIH Intramural Sequencing Center (n = 94 total including 52: accession number PRJNA448733). Blood samples were collected from healthy dogs without any underlying diseases. A total of 52 canids were included in the control group, with breed specifics of each dog are listed in the Supporting information S2 Table.

### WGS with tumor tissues in tumor groups

WGS analysis was conducted by Macrogen Inc. (Seoul, South Korea) on the tumor tissues of four dogs diagnosed with urothelial carcinoma, adenocarcinoma, rhabdomyosarcoma, and chondrosarcoma. For library construction, DNA was extracted from the tissue samples. A sequencing library of samples that passed quality control (QC) was prepared by random fragmentation of the DNA sample, followed by 5’ and 3’ adapter ligation. Adapter-ligated fragments were then amplified and gel-purified by polymerase chain reaction (PCR). The library was loaded into a flow cell for cluster generation, where fragments were captured on a lawn of surface-bound oligos complementary to the library adapters. Each fragment was amplified into distinct clonal clusters using bridge amplification. When cluster generation was complete, the templates were sequenced. After converting the sequencing data into raw data, raw reads were subjected to quality control analysis. The overall quality of the generated reads, the total number of bases, reads, genomic DNA base composition (GC) content, and basic statistics were calculated. Adapter trimming and quality filtering were performed to reduce bias in the analysis. The quality of filtered reads, total bases, total reads, GC (%), and basic statistics were calculated. After filtering the data, reads within the normal range were mapped to a reference genome (ROS_Cfam_1.0) using a bowtie2 (v2.3.5.1) [15]. After mapping, SAMTools (v1.9) and GATK (v4.1.4.0) were used to sort reads and identify variants [16,17]. The variants were classified based on each chromosome or scaffold, and information on their location was marked.

To determine annotation information, such as amino acid changes by variants, SnpEff (v4.3t) was used [18]. Since genes usually have multiple transcripts, a single variant can affect different transcripts differently. In this case, SnpEff arranges the effects of a putative sorting order considering the impact of the variants. The “most deleterious” one is shown first. Results are categorized by “impact”: high, moderate, low, and modifier. The term “high impact” refers to a variant assumed to have a high (disruptive) impact on the protein, probably causing protein truncation and loss of nonsense-mediated decay, and includes an exon-loss variant, duplication, inversion, frameshift variant, feature ablation, gene fusion, bidirectional gene fusion, rearrangement at the DNA level, protein-protein contact, structural interaction variant, rare amino acid variant, splice acceptor variant, splice donor variant, stop-lost, start-lost, and stop-gained. The term “moderate impact” refers to a non-disruptive variant that may alter protein effectiveness and includes in-frame insertion, disruptive in-frame insertion, in-frame deletion, disruptive in-frame deletion, duplication, missense variant, splice region variant, 3-prime-UTR-truncation with exon loss, and 5-prime-UTR-truncation with an exon loss variant. Transcripts were selected based on information regarding neighboring genes. The term “low impact” refers to a variant assumed to be mostly harmless or unlikely to change protein behavior, and the term “modifier impact” refers to non-coding variants or variants affecting non-coding genes where predictions are difficult or there is no evidence of impact.

### WGS with peripheral whole blood in healthy dogs

Previous studies referred to all the control group’s WGS and bioinformatics analysis methods [19]. The WGS data used in this study were obtained from the SRA (http://www.ncbi.nlm.nih.gov/sra; n = 52 unique individuals), contributed by collaborators (n = 128) or generated by the NIH Intramural Sequencing Center (n = 94 total including 52: accession number PRJNA448733). Domestic and wild canid data deposited in the SRA before April 2017 were used in this study. All Biosample numbers for the 52 genomes are listed in the Supporting information S2 Table, and the entire genome dataset can be found on NCBI [http://www.ncbi.nlm.nih.gov/bioproject/PRJNA448733]. Using the bowtie2, the dataset was mapped to a reference genome (ROS_Cfam_1.0) [15]. After alignment and variant calling, the low-quality samples were removed, e.g., samples with less than 2x the average depth, those containing corrupt data, or those found to be duplicate individuals using the ‘genome’ function in the plink version. The final dataset consisted of three wild canines and 49 purebred dogs. The complete dataset (a VCF file containing 91 million variants and 722 genomes) is available in the NCBI for Biotechnology Information database.

### Selecting SNVs in carcinoma and sarcoma groups

Bioinformatics analysis of tumor tissues was performed as follows. First, SNVs with at least one alternative allele were selected from each of the four tumor tissues in the VCF file. SNVs with a tumor minor allele frequency of 0% in the control group were further filtered, and the results are found in supporting information S3 Table. Only variants shared within each group (carcinoma and sarcoma) were selected. SNVs with a moderate-to-high impact in the SnpEff annotation file were selected and listed in supporting information in the S4A and S4B Table.

### Selecting SNVs in well-known oncogenes in four solid tumors

In each tumor sample, SNVs occurring in well-known human oncogenes were examined. Candidate genes were selected based on the existing human literature as follows: *ABL1, ALK, APC, ATM, BCL2, BCL6, BRAF, BRCA1, BRCA2, CDK4, CDKN2A, EGFR, ERBB2, FBXW7, FGFR1, FGFR2, FGFR3, JAK2, KIT, KRAS, MET, NF1, NOTCH1, TP53, CTNNB1, PDGFRβ, PDGFB, PTEN, RB1, RET, SMAD4*. These genes are commonly mutated in human cancers and are targeted by commercially available oncology panels [8,20]. First, SNVs with at least one alternative allele were selected from each of the four tumor tissues in the VCF file, and only variants that occurred within the above oncogenes were selected. SNVs with a tumor minor allele frequency of 0% in the control group were selected, and the results are listed in supporting information S5 Table. SNVs with a moderate-to-high impact in the SnpEff annotation file were selected.

### Validation of selected SNVs in publicly available carcinoma and sarcoma genomes

To validate the SNVs identified in the carcinoma group and sarcoma group, we downloaded and utilized WGS data from 3 urothelial carcinoma samples (accession number PRJNA1007700) and 2 osteosarcoma samples (accession number PRJNA525883) from NCBI. These samples were aligned to the reference genome (ROS_Cfam_1.0) using Burrows-Wheeler Aligner, and variants were identified using GATK (v4.1.4.0) [17,21]. Details of each dog are given in supporting information S6A and S6B Table.

### GO terms and KEGG pathway enrichment analysis

Gene Ontology (GO) [22] is the most widely used database in enrichment analysis. It is a helpful method for annotating genes and gene sets with biological characteristics for high-throughput genome or transcriptome data. The Kyoto Encyclopedia of Genes and Genomes (KEGG) pathway [23] is a knowledge base for systematically analyzing gene functions. GO and KEGG pathway enrichment analyses were performed using the “ShinyGO” web server [24], a Shiny application developed based on several R/Bioconductor packages. A false discovery rate (FDR) adjusted p-value cut-off of 0.05 was set as the cut-off criterion for extracting the top 20 enriched GO terms, including those for biological processes (BP), cellular components (CC), molecular functions (MF), and KEGG pathways. The top pathways were initially selected by FDR and sorted by Fold Enrichment [24].

The process for selecting a gene list for pathway enrichment analysis was as follows. First, SNVs with at least one alternative allele were selected from each of the four tumor tissues in the VCF files. SNVs with a tumor minor allele frequency of 0% in the control group were further filtered. Only the SNVs shared within the carcinoma and sarcoma groups were selected. Among the genes where the selected SNVs occurred, only those with a missing rate in the control group smaller than 2% were selected for pathway enrichment analysis [25].

## Results

### WGS analysis of tissue samples in dogs with four solid tumors

Total reads for the four dogs were as follows: dog 1, 554,475,046; dog 2, 543,095,722; dog 3, 608,789,620; dog 4, 580,040,422. The sequenced reads were aligned to the reference genome ROS_Cfam1.0 with an average alignment rate of 99.80% and an average sequencing depth of 30X. The number of transitions in which purine or pyrimidine bases were interchanged and the number of transversions between purine and pyrimidine bases was as follows: for dog 1, 2,529,031 transitions and 1,156,784 transversions; for dog 2, 2,697,705 transitions and 1,328,045 transversions; for dog 3, 2,712,974 transitions and 1,241,241 transversions; and for dog 4, 2,596,289 transitions and 1,186,984 transversions.

As a result of SnpEff annotation, intron and intergenic region variants with the highest frequency in all four tumor tissues were identified, and most other modifier variants accounted for the majority (upstream and downstream gene variants, 3-prime-UTR variants, synonymous variants, etc.) The WGS results for the four dogs included in this study are summarized in Table 3, and the results of SnpEff annotation were listed in Table 4.

**Table 3:** The results of whole genome sequencing analysis in four solid tumors.

Dog	Age	Sex	Breeds	Total reads	Mapped reads	Mean depth	SNPs	Insertions	Deletions	Transition	Transversion
**1**	12yrs	F	Dachshund	554,475,046	553,698,261 (99.86%)	30.31	3,685,815	403,957	497,016	2,529,031	1,156,784
**2**	11yrs	NM	Spitz	543,095,722	541,129,710 (99.64%)	27.13	4,025,750	437,195	419,373	2,697,705	1,328,045
**3**	5yrs	M	Poodle	608,789,620	607,929,374 (99.86%)	32.81	3,954,215	450,133	546,997	2,712,974	1,241,241
**4**	8yrs	M	Borzoi	580,040,422	579,143,074 (99.85%)	31.34	3,783,273	428,612	526,304	2,596,289	1,186,984

**Table 4:** The results of SnpEff annotation in four solid tumors.

Dog 1			Dog 2		
Type of annotation	Count	Ratio	Type of annotation	Count	Ratio
Intron variant	1,838,649	40.96%	Intergenic region	2,091,490	43.73%
Intergenic region	1,778,972	39.63%	Intron variant	1,853,744	38.76%
Upstream gene variant	523,335	11.66%	Upstream gene variant	492,422	10.30%
Downstream gene variant	243,914	5.43%	Downstream gene variant	228,616	4.78%
3_prime_UTR_variant	35,309	0.79%	3_prime_UTR_variant	34,532	0.72%
Synonymous variant	16,636	0.37%	Synonymous variant	18,817	0.39%
Intragenic variant	15,432	0.34%	Intragenic variant	17,586	0.37%
Missense variant	12,295	0.27%	Missense variant	16,050	0.34%
5_prime_UTR_variant	9,723	0.22%	5_prime_UTR_variant	11,632	0.24%
Non-coding transcript exon variant	4,893	0.11%	Non-coding transcript exon variant	6,636	0.14%
**Dog 3**			**Dog 4**		
**Type of annotation**	**Count**	**Ratio**	**Type of annotation**	**Count**	**Ratio**
Intron variant	1,986,877	40.94%	Intron variant	1,900,495	40.83%
Intergenic region	1,944,680	40.07%	Intergenic region	1,856,280	39.88%
Upstream gene variant	551,565	11.37%	Upstream gene variant	538,412	11.57%
Downstream gene variant	260,364	5.37%	Downstream gene variant	251,653	5.41%
3_prime_UTR_variant	36,556	0.75%	3_prime_UTR_variant	36,201	0.78%
Synonymous variant	17,163	0.35%	Synonymous variant	17,027	0.37%
Intragenic variant	16,868	0.35%	Intragenic variant	16,344	0.35%
Missense variant	12,682	0.26%	Missense variant	12,804	0.28%
5_prime_UTR_variant	9,959	0.21%	5_prime_UTR_variant	10,230	0.22%
Non-coding transcript exon variant	5,422	0.11%	Non-coding transcript exon variant	5,189	0.11%

**Table:** 

	Base change count												
**Dog**	**Reference**	**A**			**C**			**G**			**T**		
	**Alternative**	**T**	**G**	**C**	**A**	**T**	**G**	**A**	**T**	**C**	**A**	**G**	**C**
**1**		157,771	617,531	143,286	146,012	645,766	130,562	649,153	146,608	130,395	158,132	144,018	616,581
**2**		179,865	666,394	163,050	172,021	682,519	149,084	684,096	172,520	148,838	179,446	163,221	664,696
**3**		169,910	660,704	153,927	157,101	695,280	139,325	697,207	157,836	138,796	170,265	154,081	659,783
**4**		161,749	634,152	147,441	150,264	663,388	133,513	665,854	150,799	133,584	161,971	147,663	632,895

F, female; NM, neutered male; M, male; SNP, single-nucleotide polymorphism

### Mapping reference genome (ROS_Cfam1.0) and 52 control canids, and variant calling & selection of SNVs in tumor groups

Genes with the selected SNVs are associated with various functions. Still, notably, moderate-to-high impact mutations have been observed in a subset of genes related to immune and tumor metastatic functions. This study focused on the variants with these functions within each tumor group.

#### Carcinoma group.

Forty-three SNVs with moderate-to-high impact were identified in the carcinoma group and were localized within specific genes. Three variants were located at the exon-intron junctions of the genes [splice acceptor variant (n = 2) and splice donor variant (n = 1)]. In contrast, most of the others occurred within exons [missense variant (n = 39) and stop-gain variant (n = 1)]. The variants were identified in tumor tissues from dogs 1 and 2, with a frequency of 0% in 52 normal canids.

Among the genes with SNVs, the genes related to immune function were *NLRP12, IFNL1, TNIP2, TECPR1, ATG2A,* and *SOGA1* (Table 5). NLRP12 has two splice acceptor mutations (*NLRP12* c.2238-2A > C, *NLRP12* c.2238-1G > C), while the *IFNL1* gene had a stop-gain mutation (*IFNL1* c.421G > A). These variants are predicted to have a high impact (disruption). The remaining four SNVs were annotated as missense variants and predicted to have a moderate (non-disruptive) effect (*TNIP2* c.53C > G, *TECPR1* c.644C > T, *ATG2A* c.3977G > C, *SOGA1* c.3027T > G).

**Table 5:** The genomic locations and predicted impacts of selected SNVs in the carcinoma group.

Carcinoma	Chr	Gene name	Transcript ID	SNV position	REF	ALT	Amino acid change	Variant impact	Annotation
**Immune system**	1	*NLRP12*	ENSCAFT00845007595.1	c.2238-2A > C	A	C	.	High	Splice acceptor variant
				c.2238-1G > C	G	C			
	1	*IFNL1*	ENSCAFT00845008465.1	c.421G > A	G	A	p.Arg141*	High	Stop-gained variant
	3	*TNIP2*	ENSCAFT00845021778.1	c. 53C > G	C	G	p.Ala18Gly	Moderate	Missense variant
	6	*TECPR1*	ENSCAFT00845016274.1	c.644C > T	C	T	p.Pro215Leu	Moderate	Missense variant
	18	*ATG2A*	ENSCAFT00845049807.1	c.3977G > C	G	C	p.Arg1326Pro	Moderate	Missense variant
	24	*SOGA1*	ENSCAFT00845046876.1	c.3027T > G	T	G	p.Gln1009His	Moderate	Missense variant
**Cell adhesion and migration**	33	*ABI3 BP*	ENSCAFT00845035193.1	c.1820T > G	T	G	p.Lys607Thr	Moderate	Missense variant
	18	*MACROD1*	ENSCAFT00845054680.1	c.1135A > C	C	G	p.Thr379Pro		
				c.1129T > C	T	C	p.Ser377Pro	Moderate	Missense variant
				c.1139A > C	A	C	p.His380Pro		
	10	*CELSR1*	ENSCAFT00845016274.1	c.8116T > C	T	C	p.Ser2706Pro	Moderate	Missense variant
	36	*PKP4*	ENSCAFT00845047555.1	c.127C > G	C	G	p.Arg43Gly	Moderate	Missense variant
					c.130C > G	C	G	p.Arg44Gly	
	28	*SORBS1*	ENSCAFT00845041402.1	c.599T > G	T	G	p.Lys200Thr	Moderate	Missense variant
	20	*PKN1*	ENSCAFT00845039351.1	c.39 + 2A > C	A	C	.	High	Splice donor variant

Chr, chromosome; SNVs, single-nucleotide variants; REF, reference; ALT, alternative; * stop-gain codon

Among the genes in which SNVs occurred, the genes related to cell adhesion and migration were *ABI3 BP, MACROD1, CELSR1, PKP4, SORBS1, and PKN1* (Table 5). The SNV detected in the *PKN1* gene was annotated as a splice donor variant and was anticipated to have a high (disruptive) impact (*PKN1* c.39 + 2A > C). The remaining eight SNVs were annotated as missense variants and predicted to have a moderate (non-disruptive) effect (*ABI3 BP* c.1820T > G, *MACROD1* 1135A > C, *MACROD1* c.1129T > C, *MACROD1* c.1139A > C, *CELSR1* c.8116T > C, *PKP4* c.127C > G, *PKP4* c.130C > G, *SORBS1* c.599T > G).

#### Sarcoma group.

Fifty-eight SNVs with moderate-to-high impact localized within specific genes were identified in the sarcoma group. Two variants were located at the exon-intron junctions of the genes [splice donor variant (n = 1) and splice region variant (n = 1)], whereas the majority of the others occurred within exons [missense variant (n = 56)]. The variants were identified in tumor tissues from dogs 3 and 4, with a frequency of 0% in 52 normal canines.

Among the genes in which SNVs occurred, those related to immune function were *IRAK4, TOM1, CCDC137, CNTF, and CMTM2* (Table 6). All SNVs were annotated as missense variants and predicted to have a moderate (non-disruptive) effect (*IRAK4* c.40A > C, *IRAK4* c.44G > C, *IRAK4* c.55G > C, *TOM1* c.1456A > G, *CCDC137* c.326G > C, *CNTF* c.196C > G, *CNTF* c.199C > G, *CNTF* c.202C > G*, CMTM2* c.421G > C, *CMTM2* c.416G > C, *CMTM2* c.556C > G, *CMTM2* c.554C > G, *CMTM2* c.431G > C).

**Table 6:** The genomic locations and predicted impacts of selected SNVs in the sarcoma group.

Sarcoma	Chr	Gene name	Transcript ID	SNV position	REF	ALT	Amino acid change	Variant impact	Annotation
**Immune system**	27	*IRAK4*	ENSCAFT00845051698.1	c.55G > C	G	C	p.Arg19Gly	Moderate	Missense variant
				c.44G > C	G	C	p.Ala15Gly		
				c.40A > C	A	C	p.Trp14Gly		
	10	*TOM1*	ENSCAFT00845017738.1	c.1456A > G	A	G	p.Trp486Arg	Moderate	Missense variant
	9	*CCDC137*	ENSCAFT00845017963.1	c.326G > C	G	C	p.Ala109Gly	Moderate	Missense variant
	18	*CNTF*	ENSCAFT00845032335.1	c.202C > G	C	G	p.Ala68Pro	Moderate	Missense variant
					c.199C > G	C	G	p.Ala67Pro	
					c.196C > G	C	G	p.Ala66Pro	
	5	*CMTM2*	ENSCAFT00845020792.1	c.421G > C	G	C	p.Arg141Gly	Moderate	Missense variant
					c.416G > C	G	C	p.Ala139Gly	
					c.556C > G	C	G	p.Ala186Pro	
					c.554C > G	C	G	p.Arg185Pro	
					c.431G > C	G	C	p.Ala144Gly	
**Cell adhesion and migration**	33	*ABI3 BP*	ENSCAFT00845035193.1	c.1820T > G	T	G	p.Lys607Thr	Moderate	Missense variant
	18	*MACROD1*	ENSCAFT00845054680.1	c.1135A > C	C	G	p.Thr379Pro		
				c.1129T > C	T	C	p.Ser377Pro	Moderate	Missense variant
				c.1139A > C	A	C	p.His380Pro		
	3	*AFAP1*	ENSCAFT00845015714.1	c.757C > T	C	T	p.Gly253Arg	Moderate	Missense variant
	9	*CORO6*	ENSCAFT00845020690.1	c.28C > G	C	G	p.Ala10Pro	Moderate	Missense variant
	32	*TSPAN5*	ENSCAFT00845041510.1	c.21 + 2A > C	A	C	.	High	Splice donor variant
	4	*ADAMTS12*	ENSCAFT00845002573.1	c.1225A > C	A	C	p.Asn409His	Moderate	Missense variant
	24	*ADAM33*	ENSCAFT00845025732.1	c.2164A > C	A	C	p.Thr722Pro	Moderate	Missense variant
	18	*EXT2*	ENSCAFT00845011350.1	c.2422G > C	G	C	p.Arg808Gly	Moderate	Missense variant

Chr, chromosome; SNVs, single nucleotide variants; REF, reference; ALT, alternative.

Among the genes in which SNVs occurred, those related to cell adhesion and migration were as follows: *ABI3 BP, AFAP1, MACROD1, CORO6, TSPAN5, ADAMTS12, ADAM33, EXT2* (Table 6). The SNV detected in the *TSPAN5* gene was annotated as a splice donor variant and predicted to have a high (disruptive) impact (*TSPAN5* c.21 + 2A > C). The remaining nine SNVs were annotated as missense variants and predicted to have a moderate (non-disruptive) effect (*ABI3 BP* c.1820T > G, *MACROD1* 1135A > C, *MACROD1* c.1129T > C, *MACROD1* c.1139A > C, *AFAP1* c.757C > T, *CORO6* c.28C > G, *ADAMTS1*2 c.1225A > C, *ADAM33* c.2164A > C, *EXT2* c.2422G > C).

To further expand our findings, we utilized the whole genome sequences of the two osteosarcoma samples (PRJNA525883) and three urothelial carcinoma samples (PRJNA1007700) publicly available on NCBI. We then aimed to investigate the presence of tumor-specific mutations identified in the current study on five additional tumor genomes. Our goal was to examine the presence of tumor-specific mutations identified in our study across five additional tumor genomes. We discovered that, of the 96 candidate variants, 5 single nucleotide variants (SNVs) (3:92482211, 3:92482217, 6:36875327, 6:36875345, 6:36875456) found in carcinoma and 2 SNVs (20:45239123, 20:46629545) found in sarcoma were also present in at least one tumor genome (S6Table). While the overlap between different cancer types was modest, these seven variants may support the validity of our approach and suggest a potential genetic predisposition to a wide range of tumor types.

### Mapping reference genome (ROS_Cfam_1.0) and 52 control canids, and variant calling & selection of SNVs in oncogenes of the solid tumors

In each tumor type, variants with moderate-to-high impact in oncogenes were identified as follows: *PDGFR-β* c.2218C > T in urothelial carcinoma, *ALK* c.2662T > A, *PDGFB* c.748G > A, *CTNNB1* c.94G > A, *ABL1* c.1884T > G in adenocarcinoma, and *NOTCH1* c.1445-2A > C, *NOTCH1* c.1445-1G > C, *NOTCH1* c.1445G > C, *GLI2* c.529G > A in rhabdomyosarcoma (Table 7). Of the three variants detected in the *NOTCH1* gene in the Rhabdomyosarcoma sample, two were annotated as splice acceptor variants (*NOTCH1* c.1445-2A > C, *NOTCH1* c.1445-1G > C) and predicted to have a high (disruptive) impact. The remaining variant was annotated as a missense variant and expected to have a moderate (non-disruptive) impact (*NOTCH1* c.1445G > C). These variants were identified in 52 normal canids with a 0% frequency and were all found to be heterozygous mutations.

**Table 7:** Identified SNVs in oncogenes from four solid tumors.

Tumor	Chr	Gene name	Transcript ID	SNV position	REF	ALT	Amino acid change	Variant impact	Annotation
Urothelial carcinoma	4	*PDGFR-β*	ENSCAFT00845014520.1	c.2218C > T	C	T	p.Arg740Cys	Moderate	Missense variant
Adenocarcinoma	17	*ALK*	ENSCAFT00845020686.1	c.2662T > A	T	A	p.Asn888Tyr	Moderate	Missense variant
	10	*PDGFB*	ENSCAFT00845038929.1	c.748G > A	G	A	p.Val250Ile	Moderate	Missense variant
	23	*CTNNB1*	ENSCAFT00845031553.1	c.94G > A	G	A	p.Asp32Asn	Moderate	Missense variant
	9	*ABL1*	ENSCAFT00845036288.1	c.1884T > G	T	G	p.Lys628Asn	Moderate	Missense variant
Rhabdomyosarcoma	9	*NOTCH1*	ENSCAFT00845009762.1	c.1445-2A > C	A	C	.	High	Splice acceptor variant
				c.1445-1G > C	G	C		High	Splice acceptor variant
				c.1445G > C	G	C	p.Gly482Ala	Moderate	Missense variant
	19	*GLI2*	ENSCAFT00845047496.1	c.529G > A	G	A	p.Arg177Trp	Moderate	Missense variant

Chr, chromosome; SNVs, single nucleotide variants; REF, reference; ALT, alternative

### GO terms and KEGG pathway enrichment analysis in tumor groups

Pathway Enrichment Analysis (GO and KEGG) was conducted to determine whether pathways highly relevant to immune system function, cell adhesion, and migration function have been enriched by SNVs shared within a group.

#### Pathway enrichment analysis in the carcinoma group.

**Fig 1:**
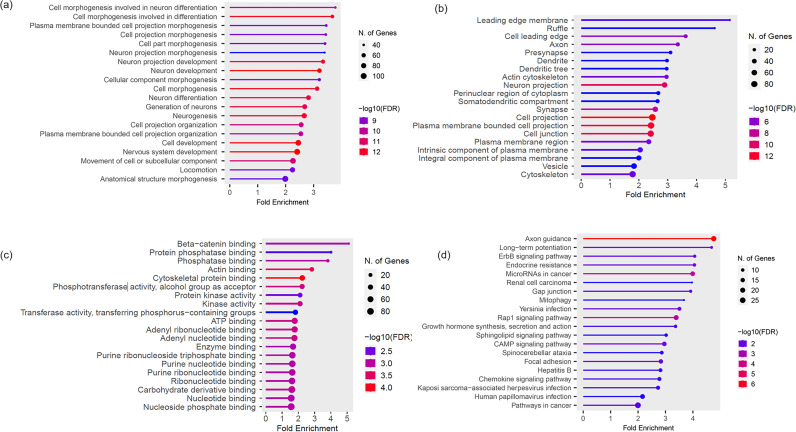
Gene Ontology (GO) and Kyoto Encyclopedia of Genes and Genomes (KEGG) pathway of carcinoma group. **(A)** GO (biological process; BP) **(B)** GO (cellular component; CC) **(C)** GO (molecular functions; MF) **(D)** KEGG.

A total of 587 genes with shared SNVs in the tumors of the carcinoma group were subjected to GO term enrichment and KEGG pathway analyses. The top 20 enriched GO terms (BP, CC, and MF) and KEGG pathways of the carcinoma group are shown in Fig 1.

GO (BP) revealed only general biological pathways, whereas GO (CC) and GO (MF) were significantly enriched in the pathways relevant to cell adhesion and migration. According to the results, the enriched CC terms were actin cytoskeleton (GO:0015629), cell junction (GO:0030054), cytoskeleton (GO:0005856) and MF terms were beta-catenin binding (GO:0008013), protein phosphatase binding (GO:0019903), phosphatase binding (GO:0019902), actin binding (GO:0003779), cytoskeletal protein binding (GO:0008092) (Table 8). KEGG pathways revealed pathways relevant to immune function, cell adhesion, and migration. The enriched KEGG pathways included the chemokine signaling pathway (cfa04062), ErbB signaling pathway (cfa04012), gap junction (cfa04540), rap1 signaling pathway (cfa04015), and focal adhesion (cfa04510) (Table 9).

**Table 8:** GO terms relevant to cell adhesion and migration function in the carcinoma group.

GO	GO ID	Pathway name	Gene number	Genes	Enrichment FDR	Fold Enrichment
**CC**	GO:0015629	Actin cytoskeleton	32	*PTK2, DAPK1, PLEKHH2, MYH14, COBL, LCP1, PAK1, MOBP, DPYSL3, FAM107A, FER, PLS1, FMN1, LIMA1, EPHA3, SRC, MYO1B, PKNOX2, ARHGAP6, ACTC1, NF2, MYO3B, HDAC4, CAPZB, ACTR10, SRCIN1, MYH8, MYO15A, MYO10, ABL1, DMD, GMFG*	0.00000357	2.958530914
	GO:0030054	Cell junction	84	*DCC, KCNC2, NCAPH2, PRKN, THEMIS, PTK2, PACSIN1, EXOC4, NTRK2, JAK2, ZAP70, CHRM2, CNTNAP2, CADPS2, CDK5RAP2, PARD3, PPFIA3, ITGB1, GRIN2D, GAD2, GRM5, LCP1, FRMD4A, EPB41L5, PAK1, PDE2A, SYT1, PLCB1, MAGI1, RAB3C, FAM107A, FER, EFNA5, ERC2, FCHO2, LIMA1, SORBS1 RAPGEF2, SRC, RPH3A, CDH17, CTNND2, FRMPD4, PECAM1, CBLN4, ACTC1, TNS3, NF2, HDAC4, EPS8, STX6, PARD3B, MAGI3, SPG11, IL1RAPL1, ITGB3, DRD2, LRFN5, CDH23, TNR EPHB2, TNN, SORCS2, DNM3, NLGN1, NRG3, SRCIN1, SKAP1 CACNA1A, STX10, STON2, ARHGAP44, VAV1, GABRA3, PDPK1, GRIN1, NPHP4, DMD, CNTN2, GABRA2, HTR2A, VWC2L*	0.00000000000828	2.409751968
	GO:0005856	Cytoskeleton	84	*TTLL8, PACRG, PTK2, PACSIN1, DAPK1, DNAI1, KANK1, JAK2, PLEKHH2, PRKCE, DNAH11, EML6, RRAGD, MYH14, COBL, GLI3, CDK5RAP2, CFAP100, AGBL4, STIL, DEUP1, RB1, LCP1, TPT1, FRMD4A, GLI2, EPB41L5, PAK1, MOBP, FNTA, ANK1, NAV3, DPYSL3, SNTG1, FAM107A, FER, RILPL1, PLS1, DNAH6, ERC2, FCHO2, FMN1, LIMA1, SORBS1, MCPH1, EPHA3, SRC, AMBRA1, MYO1B, PKNOX2, ARHGAP6, FRMPD4, YEATS2, ADCY5, ACTC1, NF2, MYO3B, HDAC4, SNX4, KIFBP, CDH23, DNM3, CAPZB, ACTR10, TMEM63A, SRCIN1, CEP128, KLHL4, MYH8, CLTC, DNAH9, EML1, CCP110, MYO15A, DNAI4, MYO10, NPHP4, ABL1, USP10, DMD, GMFG, RND3, FBXL7*	0.00000777	1.781183273
**MF**	GO:0008013	Beta-catenin binding	10	*PRKN, EP300, KANK1, GLI3, CHD8, TCF7L1, MED12L, PTPRT, CTNND2, RORA*	0.001329	5.098182736
	GO:0019903	Protein phosphatase binding	11	*PPP6R2, PTK2, PPP6R1, ANK1, FER, SOD1, PPP1R16B, KCNQ1, PHACTR3, SKAP1, TRAF3*	0.004353445	4.005715007
	GO:0019902	Phosphatase binding	14	*PPP6R2, PTK2, CHCHD3, PPP6R1, ANK1, FER, SOD1, PPP1R16B, KCNQ1, NFATC2, PHACTR3 CNST, SKAP1, TRAF3*	0.001114073	3.813907696
	GO:0003779	Actin binding	29	*PHACTR2, PRKN, PACRG, PTK2, PLEKHH2, PRKCE, MYH14, COBL, ITGB1, LCP1, MOBP, MICAL2, PLS1, LIMA1, JMY, MYO1B, PKNOX2, PHACTR3, NF2, MYO3B, EPS8, CAPZB, KCNMA1, MYH8, MYO15A, MYO10, ABL1, DMD, GMFG*	0.000244985	2.827680589
	GO:0008092	Cytoskeletal protein binding	50	*PHACTR2, PRKN, PACRG, PTK2, PACSIN1, AGTPBP1, PLEKHH2, PRKCE, MYH14, COBL, CDK5RAP2, ITGB1, AGBL4, SPATA6, RAB10, LCP1, EPB41L5, PAK1, MOBP, TRAK1, FNTA, ANK1, NAV3, DPYSL3, RAB3C, MICAL2, PLS1, FMN1, LIMA1, JMY, PTPRT, MYO1B, UNC5C, PKNOX2, ACTC1, PHACTR3, NF2, MYO3B, EPS8, KIFBP, DNM3, CAPZB, KCNMA1, MYH8, EML1, MYO15A, MYO10, ABL1, DMD, GMFG*	0.0000809	2.241663263

GO, Gene Ontology; CC, cellular component; MF, molecular functions; FDR, false discovery rate

**Table 9:** KEGG pathways relevant to the immune system, along with cell adhesion and migration function, in the carcinoma.

KEGG	KEGG ID	Pathway name	Gene number	Genes	Enrichment FDR	Fold Enrichment
cfa04062	Chemokine signaling pathway	13	*PTK2, GNAQ, JAK2, PARD3, BRAF, PAK1, PLCB1, SRC, PREX1, ADCY5, VAV1, CCL13*	0.018637439	2.778051671
cfa04012	ErbB signaling pathway	9	*PTK2, BRAF, PAK1, SRC, MAPK10, NRG3, MAP2K2, ABL1*	0.011028532	4.065639397
cfa04540	Gap junction	9	*GNAQ, GRM5, PLCB1, SRC, ADCY5, DRD2, MAP2K2, HTR2A PRKG1*	0.013124162	3.916896492
cfa04015	Rap1 signaling pathway	19	*GNAQ, RAPGEF5, PARD3, ITGB1, BRAF, PLCB1, MAGI1, EFNA5, RAPGEF2, SRC, ADCY5, MAGI3, ITGB3, DRD2, SKAP1, VAV1, MAP2K2, GRIN1*	0.000349297	3.390291519
cfa04510	Focal adhesion	15	*PTK2, PIP5K1B, ITGB1, BRAF, PAK1, SRC, MAPK10, COL6A3, ITGB3, TNR, TNN, LAMA3, VAV1, PDPK1*	0.010184312	2.832323742

KEGG, Kyoto Encyclopedia of Genes and Genomes; FDR, false discovery rate

### Pathway enrichment analysis in the sarcoma group

**Fig 2:**
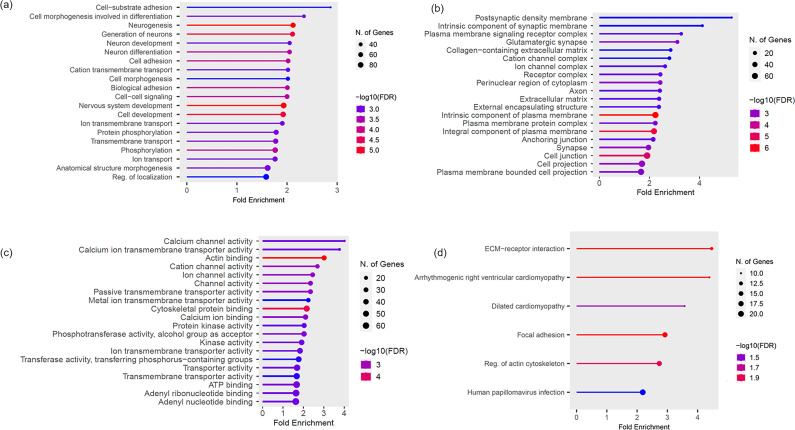
Gene Ontology (GO) and Kyoto Encyclopedia of Genes and Genomes (KEGG) pathway of sarcoma group. **(A)** GO (biological process; BP) **(B)** GO (cellular component; CC) **(C)** GO (molecular functions; MF) **(D)** KEGG.

A total of 641 genes with commonly occurring SNVs in the tumors of the sarcoma group were subjected to GO term enrichment and KEGG pathway analyses. The top 20 enriched GO terms (BP, CC, and MF) and KEGG ontology terms of the sarcoma group are shown in Fig 2.

GO (BP), GO (CC), and GO (MF) analyses were all significantly enriched in pathways relevant to cell adhesion and migration. According to the results, the enriched BP terms were cell-substrate adhesion (GO:0031589), cell adhesion (GO:0007155), biological adhesion (GO:0022610), cell-cell signaling (GO:0007267), and CC terms were collagen−containing extracellular matrix (GO:0062023), receptor complex (GO:0043235), extracellular matrix (GO:0031012), anchoring junction (GO:0070161), cell junction (GO:0030054). The MF terms were actin binding (GO:0003779) and cytoskeletal protein binding (GO:0008092) (Table 10). KEGG pathways revealed pathways relevant to cell adhesion and migration. Enriched KEGG pathways included ECM-receptor interaction (cfa04512) and focal adhesion (cfa04510) (Table 11).

**Table 10:** GO terms relevant to cell adhesion and migration function in the sarcoma group.

GO	GO ID	Pathway name	Gene number	Genes	Enrichment FDR	Fold Enrichment
**BP**	GO:0031589	Cell-substrate adhesion	22	*PTK2, PRKCE, LAMB4, FBLN2, JAG1, FAM107A, PARVA, FMN1, TIAM1, ABI3 BP, NEDD9, ITGB6, PREX1, ITGB5, COL26A1, TNN CASK, ITGAV, ACTN1, SRCIN1, ITGA11, SPOCK1*	0.002285933	2.869213187
	GO:0007155	Cell adhesion	59	*PTK2, ZAP70, SHB, PRKCE, IL4I1, LAMB4, FBLN2, SDK2, DLG2, TENM4, ITGA9, CYTH1, C1QTNF1, JAG1, CNTN6, UNC5D, FAM107A, CLSTN2, PARVA, ROBO1, ROBO2, FMN1, SPON1, PAG1, CX3CL1, TIAM1, ABI3 BP, NFKBIZ, NEDD9, ITGB6, CTNND2, KIRREL3, PREX1, LEF1, PECAM1, CASS4, COL6A3, CDH26, ITGB5, COL26A1, ASTN1, TNR, EPHB2, TNN, CASK, ITGAV, KIF26B, ITPKB, PCDH7, ACTN1, SRCIN1, ITGA11, VAV1, LAMA1, CDH11, ADGRG1, PRKG1, SPOCK1*	0.000172209	2.021078391
	GO:0022610	Biological adhesion	59	*PTK2, ZAP70, SHB, PRKCE, IL4I1, LAMB4, FBLN2, SDK2, DLG2, TENM4, ITGA9, CYTH1, C1QTNF1, JAG1, CNTN6, UNC5D, FAM107A, CLSTN2, PARVA, ROBO1, ROBO2, FMN1, SPON1, PAG1, CX3CL1, TIAM1, ABI3 BP, NFKBIZ, NEDD9, ITGB6, CTNND2, KIRREL3, PREX1, LEF1, PECAM1, CASS4, COL6A3, CDH26, ITGB5, COL26A1, ASTN1, TNR, EPHB2, TNN, CASK, ITGAV, KIF26B, ITPKB, PCDH7, ACTN1, SRCIN1, ITGA11, VAV1, LAMA1, CDH11, ADGRG1, PRKG1, SPOCK1*	0.000172209	2.006264204
	GO:0007267	Cell-cell signaling	60	*PRKN, PLA2G6, SLC24A2, CDK14, RFX3, CLOCK, TFAP2B, ROR2, GABBR2, PRKCE, ABCA1, CRHR2, GRIK3, CFTR, PANX1, DLG2, NAPB, RBMS3, ZEB2, C1QTNF1, CACNB4, UBAC2, CHD7, PXK, CLSTN2, ERC2, DHH, NKD1, CTNND2, ZNF423, BRSK2, RYR2, TERT, LEF1, ADCY5, KALRN, STX1A, CTBP2, TNR, EPHB2, TNN, CASK, SORCS2, NRG3, GRID1, DAGLA, DLGAP2, CACNA1A, PIN1, SREBF1, NOS2, GRIN2A, MECP2, NPHP4, JPH3, WWOX, NIBAN2, CDH11, GABRA2, CACNG2*	2.00046878	0.000172209
**CC**	GO:0062023	Collagen−containing extracellular matrix	15	*LAMB4, FBLN2, COL4A2, FRAS1, FMOD, ABI3 BP, NAV2, COL6A3, FGFR2, TNR, TNN, CASK, ANXA2, LAMA1, GPC6*	0.00907007	2.848620748
	GO:0043235	Receptor complex	24	*ROR2, ZAP70, GABBR2, GRIK3, DLG2, ITGA9, LRP1B, PTPRQ, ITGB6, NTRK3, FGFR2, ITGB5, EPHB2, ITGAV, CALCRL, IL6R, ITGA11, IL23R, PRLR, GRIN2A, TGFBR3, GABRA2, HTR2A, CACNG2*	0.003384754	2.43557074
	GO:0031012	Extracellular matrix	23	*CCBE1, COL22A1, LAMB4, FBLN2, COL4A2, SPON1, MMP15, FRAS1, HPSE2, FMOD, ABI3 BP, NAV2, ADAMTS17, COL6A3, FGFR2, COL26A1, TNR, TNN, CASK, MMP3, ANXA2, LAMA1, GPC6*	0.00455181	2.386288691
	GO:0070161	Anchoring junction	30	*PTK2, ZAP70, PANX1, DLG2, FRMD4A, JAG1, SIRT2, MAGI1, FAM107A, PARVA, TIAM1, TWF1, ITGB6, KIRREL3, SCN2A, PECAM1, ITGB5, HEG1, ANK3, LPP, CASK, AFAP1, ITGAV, ACTN1, ITGA11, VAV1, LAMA1, NPHP4, NIBAN2, CDH11*	0.003959735	2.15061434
	GO:0030054	Cell junction	73	*PPM1H, PRKN, QKI, PTK2, ZAP70, SPTBN1, CRHR2, GRIK3, CDK5RAP2, PANX1, TSPAN1, SDK2, DLG2, FRMD4A, ATP2B2, NAPB, JAG1, SIRT2, CACNB4, CNTN6, DCLK1, MAGI1, FAM107A, CLSTN2, PARVA, ERC2, RAPGEF2, TIAM1, TWF1, ITGB6, CTNND2, CAP2, KIRREL3, SCN2A, PECAM1, CBLN4, NLRX1, ITGB5, HEG1, STX1A, ANK3, CTBP2, MACO1, LPP, TNR, EPHB2, TNN, CASK, SORCS2, AFAP1, ITGAV, NRG3, GRID1, SLC29A4, DAGLA, DLGAP2, ACTN1, SRCIN1, CACNA1A, STX10, ITGA11, TSPOAP1, PIN1, VAV1, LAMA1, GRIN2A, MECP2, NPHP4, NIBAN2, CDH11, GABRA2, HTR2A, CACNG2*	0.000015	1.905644844
**MF**	GO:0003779	Actin binding	34	*PHACTR2, PRKN, PTK2, LIMCH1, CORO2A, PRKCE, MYO6, SPTBN1, MYH14, COBL, PANX1, MYO3A, PXK, MICAL2, PARVA, PLS1, ROBO2, JMY, TWF1, CAP2, PKNOX2, PHACTR4, MLPH, MYOZ2, MYO3B, ARPC1B, KCNMA1, SPTB, MICAL3, ACTN1, GAS2L2, MYO15A, MYH11, RAI14*	0.0000104	3.016736071
	GO:0008092	Cytoskeletal protein binding	53	*PHACTR2, PRKN, PTK2, LIMCH1, CORO2A, PRKCE, MYO6, SPTBN1, MYH14, COBL, CDK5RAP2, CNTRL, AGBL4, PANX1, MYO3A, ANK1, MYBPC1, PXK, MICAL2, PARVA, PLS1, ROBO2, DIP2B, FMN1, PACSIN3, JMY, PTPRT, TWF1, CAP2, KIF13A, PKNOX2, PHACTR4, MLPH, MYOZ2, MYO3B, ANK3, EFHC2, ARPC1B, KCNMA1, KIF26B, SPTB, MICAL3, ACTN1, ANXA2, EML5, BCAS3, GAS2L2, MYO15A, MYH11, SGIP1, RAI14, NEK6, TTLL11*	0.0000539	2.16223198

GO, Gene Ontology; BP, biological process; CC, cellular component; MF, molecular functions; FDR, false discovery rate

**Table 11:** KEGG pathways relevant to cell adhesion and migration function in the sarcoma group.

KEGG	KEGG ID	Pathway name	Gene number	Genes	Enrichment FDR	Fold Enrichment
cfa04512	ECM-receptor interaction	11	*LAMB4, ITGA9, FRAS1, ITGB6, COL6A3, ITGB5, TNR, TNN, ITGAV, ITGA11, LAMA1*	0.008108869	4.465213023
cfa04510	Focal adhesion	17	*PTK2, PIP5K1B, LAMB4, ITGA9, PARVA, ITGB6, PIK3CA, COL6A3, ITGB5, TNR, TNN, ITGAV, ACTN1, ITGA11, VAV1, LAMA1, VAV2*	0.008108869	2.920966672

KEGG, Kyoto Encyclopedia of Genes and Genomes; ECM, extracellular matrix; FDR, false discovery rate

## Discussion

Canine solid tumors were classified into two groups, carcinomas and sarcomas, to investigate the common mechanisms that influence tumor formation within each group. Consequently, many SNVs and altered GO (BF, CC, and MF) and KEGG pathways influencing functions associated with the immune system or tumor metastasis were determined. Although mutations related to immune function were similarly observed in both groups, distinctions between the two tumor groups were noted for mutations associated with tumor metastatic function. Mutations were identified in genes encoding cell adhesion molecules in the carcinoma group, whereas significant variations in genes encoding extracellular matrix (ECM)-related molecules were observed in the sarcoma group.

First, in terms of immune function, variations related to the nuclear factor kappa B (NF-κB) signaling pathway (*NLRP12, TNIP2, IRAK4*) and the autophagy process (*TECPR1, ATG2A, SOGA1, TOM1*) were identified in both carcinoma and sarcoma tumor groups. Tumors are generally considered to be deeply connected with the immune system. Indeed, many tumors are known to have the ability to evade immunity or create an immunosuppressive tumor microenvironment. Additionally, chronic inflammation can serve as a cause of cancer development [26,27]. Identifying pathways associated with immune-related mutations is a necessary step for developing cancer therapies targeting those pathways. The NF-κB signaling pathway is a critical mediator for inflammatory response in tumors, and inhibiting this pathway in tumor cells often reduces tumor size [28,29]. High-impact mutations in the *NLRP12* gene were also identified in the carcinoma group. *NLRP12* encodes a protein that is a negative regulator of non-canonical NF-κB signaling [30], indicating that mutations in this gene may stimulate the NF-κB pathway and contribute to tumor formation. The protein encoded by the *TNIP2* gene is also considered an important inhibitor of the NF-κB pathway and is known to regulate tumor aggressiveness in various cancer types [31]. In the sarcoma group, variants of *IRAK4* were identified, which activated the NF-κB signaling pathway. High expression of this gene is associated with poor prognosis in colorectal cancer patients [32].

Autophagy, conversely, regulates cellular homeostasis by regulating the turnover and elimination of cellular components such as proteins and cell organelles [33]. Autophagy has been proposed to suppress tumors in the early phases of solid tumor formation; however, it promotes cell mobility and invasiveness in later stages [33–36]. *TECPR1*, identified in the carcinoma group, is associated with downregulating autophagy-related gene 5 (*ATG5*) when expressed at low levels in non-small cell lung cancer, suppressing autophagy and enhancing cell viability [37]. *ATG2A* is an essential core member of the autophagy machinery, and its upregulation can promote autophagy, potentially contributing to the development of tumors, such as glioblastoma and hepatocellular carcinoma [38,39]. *SOGA1* is an autophagy inhibitor, and its increased expression has been observed in various tumors, such as bladder cancer, colorectal cancer, and hepatocellular carcinoma [40,41]. *TOM1*, identified in the sarcoma group, is also a gene involved in the autophagy process, and it has been revealed that a low level of *TOM1* is associated with an increased incidence of solid tumors [42]. To consider autophagy as a therapeutic target, it is essential to understand the relative frequencies and genetic contexts of mutations that inhibit or promote autophagy within tumors [33]. In this study, mutations in various genes associated with autophagy were identified, which may contribute to the regulation of autophagy at different stages of tumor development.

These two biological pathways are recognized as mechanisms that play a role in developing various tumors, including most solid tumors and hematological malignancies in humans [33,43–45]. Despite the limited number of studies, there is evidence that similar signaling aberrations occur in canine cancers. Research suggests the overactivation of the NF-κB pathway in dogs with diffuse large B-cell lymphomas, mammary carcinoma, malignant melanoma, osteosarcoma, and prostate tumors [46]. Studies have revealed the tumor-suppressive effects of autophagy inhibitors in canine tumors, including mammary tumors and osteosarcoma [47–49]. Therefore, in the present study, these pathways were postulated as potential biological mechanisms that could be targeted in both canine carcinoma and sarcoma.

In addition to the immune-related genes, mutations and altered pathways related to cell adhesion and migration were detected in both groups. During cancer progression, cells lose their original tissue contact, move through the ECM, enter the lymphatic and/or blood system, extravasate, and ultimately form new tumors. Therefore, tumor cells inevitably experience alterations in cell-cell and cell-ECM adhesion, and changes in cell adhesion molecules and ECM components can enhance the metastatic ability of cancer cells [50]. Genes with these functions are important in cancer research because they are closely related to malignant tumors’ invasion and metastasis characteristics. Interestingly, significant differences in gene mutations and altered pathways were observed between the carcinoma and sarcoma groups. In the carcinoma group, mutations were primarily observed in genes encoding cell adhesion molecules and related pathways. In contrast, in the sarcoma group, mutations were mainly observed in genes and pathways related to ECM.

In carcinomas, the genes related to cell adhesion molecules are *MACROD1*, *CELSR1*, and *PKP4*, with a high-impact mutation detected in *PKP4*. Mutations in *MACROD1* were present in both tumor groups. This gene does not directly encode a cell adhesion molecule but encodes ADP-ribose hydrolases. Overexpression of this gene in tumors promotes invasion and metastasis by lowering epithelial cadherin (E-cadherin) expression [51]. CELSR1 is a cadherin superfamily member, and aberrant *CELSR1* expression has been observed in various tumor types, including glioma and gastric cancer [52,53]. The p0071 protein encoded by *PKP4* is a member of the armadillo protein family that constitutes desmosomes. This protein is essential for the formation and regulation of two types of cell-cell adhesions and can also modulate Rho signal transduction. Changes in Rho GTPase signaling are associated with tumor development [54,55]

Additionally, in the pathway enrichment analyses of carcinoma, altered pathways related to adherens junction molecules and desmosomes, such as beta-catenin binding (GO:0008013) in GO (MF) pathways, the ErbB signaling pathway (cfa04012) and Ras-associated protein 1 (Rap1) signaling pathway (cfa04015) in KEGG pathways, were identified. Beta-catenin is a crucial component of cell adhesion, serving as a constituent of the desmosome and a critical regulator in the Wnt signaling pathway. Dysregulation of Beta-catenin signaling is associated with tumorigenic properties [56,57]. Activation of EGFR, found in the ERBB signaling pathway, downregulates E-cadherin through various post-transcriptional mechanisms to reduce cell-cell adhesion and enhance cellular motility [58,59]. Hence, both beta−catenin binding and the ERBB signaling pathway are closely associated with regulating cell adhesion capability and increasing tumor metastatic potential [59]. In addition, the Rap1 signaling pathway regulates integrins and cadherins, which are crucial for cell adhesion to the ECM and intercellular adhesion, which could mediate cell attachment during tumor cell invasion and metastasis [60].

Loss of cell adhesion and changes in cadherin expression are common characteristics of malignant cells and indicate aggressive tumor growth and poor prognosis. In the carcinoma group, frequent mutations were identified in the cell adhesion molecules and desmosomal components. In previous human studies, the downregulation of these molecules was associated with the development of an aggressive invasive phenotype [61–63]; this is also relevant to carcinoma in dogs; for example, reduced E-cadherin expression is a common occurrence in canine mammary tumors and is associated with poor prognosis, including increased tumor proliferation and lymph node metastasis [64,65]. Consequently, in this study, we postulated that mutations in genes encoding adherens junction molecules and desmosome structural constituents might have a more specific association with carcinoma.

Conversely, genetic mutations and alterations in pathways related to ECM components were predominantly observed in the sarcoma group. The ECM is a major constituent of the tumor microenvironment, and ECM remodeling can occur in tumor tissues. These changes can potentially induce tumor metastasis and dissemination [66]. Research on the interaction between sarcomas and ECM has not yet been thoroughly conducted; however, recent evidence indicates that mutations in ECM molecules may be significant for the progression and prognosis of sarcomas [67]. In a study by Pearce et al., a high ECM protein matrix index was associated with poorer overall survival in the Cancer Genome Atlas (TCGA) sarcoma cohort [68].

The ECM component-related genes mutated in the sarcoma group were *ADAMTS12, ADAM33, TSPAN5, AB3IBP,* and *EXT2.* The ADAMTS12 protein is a member of the ADAMTS (a disintegrin and metalloproteinase with thrombospondin motifs) protein family, and all members of this gene family function as metalloproteinases that contribute to the formation, homeostasis, and remodeling of the ECM [69,70]. The protein encoded by *ADAM33* is also a significant metalloproteinase in the ECM, which is crucial for tissue remodeling [71], and the absence or low expression of this protein contributes to increased tumor aggressiveness and metastasis [72]. Tetraspanin 5, encoded by *TSPAN5*, in which a high-impact mutation has occurred, acts as a major partner of the cell adhesion molecules integrins and interacts with a wide range of ECM proteins. These interactions may be the pathways through which tetraspanins affect cell migration and metastasis [73]. Variants of *ABI3 BP* occur in both tumor groups, and this gene encodes an ECM protein that promotes cell adhesion and ECM assembly [74]. *EXT2* encodes exostosin glycosyltransferase-2, which plays an essential role in the elongation of heparan sulfate chains, a component of the ECM [75]. According to the pathway analysis, several altered pathways related to the ECM were identified compared to those in the carcinoma group. Pathways such as collagen-containing extracellular matrix (GO:0062023), receptor complex (GO:0043235), and extracellular matrix (GO:0031012) were identified in GO (CC), and ECM-receptor interaction (cfa04512) pathways were identified by KEGG analysis.

The differences observed in the mutations in molecules related to cell adhesion and migration between the carcinoma and sarcoma groups may be associated with variations in the metastatic processes occurring in each tumor type. It is generally accepted that carcinomas tend to develop lymph node metastases more frequently than sarcomas, and approximately 20–40% of cancer types cause only hematogenous metastases without lymph node involvement. Conversely, mesenchymal tumors, with the rare exceptions of clear cell sarcoma, epithelioid sarcoma, angiosarcoma, and alveolar rhabdomyosarcoma, usually favor vascular spread during the metastatic process [9,10].

During the metastatic process of carcinoma, epithelial tumor cells may undergo an epithelial-to-mesenchymal transition (EMT), in which epithelial cells adopt a more mesenchymal state and acquire the migratory and invasive characteristics of mesenchymal cells [61]. This process allows cancer cells to dissociate from the primary tumor and enter the bloodstream or lymphatic circulation, facilitating the colonization of distant sites [9,76]. Both adherens junctions and desmosomes must be disrupted for epithelial cells to dissociate during EMT [61]. This study detected mutations in genes related to cadherins and desmosomes, suggesting a potentially close association between these genetic mutations and the metastasis of epithelial tumors.

Mesenchyme-derived sarcoma cells are equipped with phenotypic features typically induced by EMT in epithelial cells and do not need to undergo EMT to acquire them [77]. When mesenchymal cells undergo metastasis, they migrate through capillaries and actively modify the metastatic soil to promote subsequent metastatic tumor growth [10]. Various cell types participate in the process of establishing a tumor microenvironment in which they secrete matrix metalloproteinases (MMPs) that, in turn, lead to ECM degradation, which is a necessary step for cancer cell invasion [78]. In the present study, mutations were identified in two genes, the *ADAMTS12* and the *ADAM33*, encoding MMPs.

In addition, a high-impact mutation in *NOTCH1* was confirmed in rhabdomyosarcoma. Active Notch signaling was shown to control rhabdomyosarcoma cell migration and invasion, which is associated with changes in the expression of adhesion molecules, including the integrin α9 subunit. Integrins play a central role in mediating cell attachment to the ECM and are essential for driving changes within the ECM [67]. Notch activation enhances the metastatic ability of osteosarcoma and is associated with hematogenous metastasis in sarcoma [10]. These findings further emphasize the relevance of ECM, hematogenous metastasis, and its association with sarcomas.

Most of our knowledge regarding ECM-tumor cell interactions has been derived from research on epithelial tumors. There is still a lack of knowledge regarding the interactions between sarcoma and ECM and how this can affect the clinical course of the disease [67,79]. In this context, the results of this study provide evidence supporting the concept that mutations in these ECM proteins can also play a significant role in the progression of canine sarcomas.

The altered biological mechanisms identified in this study, believed to play a role in tumor formation, are currently under investigation as potential therapeutic targets for cancer treatment in humans. In the case of the NF-κB signaling pathway, there are inhibitors like IκB kinase inhibitor (anti-inflammatory drugs and natural compounds such as curcumin), and Chloroquine and its analog hydroxychloroquine have been approved and used as autophagy inhibitors [46,80–82]. Furthermore, therapeutic strategies targeting cell adhesion molecules include α-solanine, which stimulates E-cadherin expression, and integrin inhibitor Intetumumab (formerly CNTO 95) [83–85]. Lastly, ongoing studies and clinical trials targeting the ECM are actively underway, including antibodies targeting ECM-associated receptors on the membranes of cancer cells and reengineered CAR-T cells that can degrade ECM components and increase T-cell infiltration [66,86].

Dogs and humans are not only exposed to similar environments but also share similarities in tumor development. For example, mutations in genes identified in canine mammary cancer are also observed in human breast cancer. A notable example is the p.H1047R hotspot mutation in the *PIK3CA* gene, which is well-known in human breast cancer and has also been found in canine mammary cancer [87]. In addition, common mutations have been observed in both canine and human tumors in genes such as *KRAS*, *NRAS*, *BRAF*, *KIT*, and *EGFR* [88]. The presence of these shared mutations suggests that the process of cancer development may be similar in dogs and humans, making canine models valuable for studying cancer and developing treatments. Furthermore, for cancers that are rare or difficult to sample in humans, similar canine tumors can be utilized for research, providing valuable insights into these types of cancers. In this study, genetic mutations in the malignant tumors of dogs were investigated, and various biological mechanisms were considered to affect tumor development and progression within each group. The SNVs identified here may not be significant mutations that drive tumor formation. However, the concept of mini-driver mutations has gained attention in recent theories of tumorigenesis. It supports the idea that multiple mutations with small selective advantages can influence tumor formation together [89]. Hence, identifying shared biological mechanisms affected by these mutations may provide a basis for applying therapies targeting the same mechanisms in veterinary clinical settings, similar to human medicine [4]. Furthermore, the differences observed between the carcinoma and sarcoma groups indicated that different therapeutic approaches are required for these two tumor types.

However, this study has several limitations. First, whole-blood-matched sequencing was not performed for each tumor sample; thus, complete exclusion of germline mutations could not be achieved. Because variants that did not occur in the control group were selected as the criteria, the probability of germline mutations was estimated to be low. However, for verification, the presence of the corresponding variants must be confirmed in germline DNA derived from the peripheral blood leukocytes of the same dog. Second, the effects of the identified variants on tumor formation were not confirmed. Additional studies employing approaches such as transcriptome analysis or animal models with identified mutations must confirm this. Third, structural variants (SVs) were not investigated. As known, pathogenic SVs used for diagnosis or therapeutic stratification have been identified in over 30% of human cancers [90]. SVs can impact more base pairs in the genome than SNVs and have more serious effects on the phenotype [91]. Therefore, additional research considering SVs should be necessary. Fourth, relevant pathways may have been missed, or the selected pathways may not have been universally applicable across various tumor types, as the number of tumor tissues included in this study was too small. Therefore, further research involving larger populations is required. It is necessary to conduct analyses on a wider variety of tumor types or a larger population to identify additional mutations and pathways and to generalize the results. Fifth, SNVs were selected, and variations with a high missing rate in the control group were included. The small number of participants (n = 52) in the control group was considered a contributing factor. Finally, in this study, we utilized ROS_Cfam_1.0, a recently developed high-quality reference genome (released on Sep 3, 2020). A single reference genome cannot fully capture all the variations present across diverse breeds and tumors, which may lead to some variations being miscalled or missed. newer reference genomes, such as CanFam4 (UU_Cfam_GSD_1.0), CanFam5 (UMICH_Zoey_3.1), and CanFam6 (Dog10K_Boxer_Tasha), also exist. According to previously published studies, when the entire CanFam3.1 genome was converted to align with more recent reference genomes, such asCanFam4 (UU_Cfam_GSD_1.0), CanFam5 (UMICH_Zoey_3.1), and CanFam6 (Dog10K_Boxer_Tasha), high mapping feasibility and low lift-over failure rates were observed. This indicates the high quality and similarity between reference genomes, suggesting that when results are generated using a single reference genome, similar outcomes are likely to be obtained with other reference genomes [92].

## Supporting information

S1 Fig Histopathologic examination from the samples of the tumors in four dogs. (A) Histopathological observation of the bladder mass (dog1), (B) intestinal mass (dog 2), nasal mass (dog 3), and the mass from the right hindlimb (dog 4). (A) It revealed highly invasive tumor that extended transmurally throughout bladder wall and disrupted the normal architecture. Neoplastic epithelial cells were polygonal with distinct cell borders and abundant eosinophilic cytoplasm. There is marked anisocytosis and anisokaryosis (H&E, × 5, inset: H&E, × 40). (B) Intestinal mucosa was regionally infiltrated and expanded by a poorly demarcated and markedly infiltrative neoplastic mass extending through intestinal wall segments. The neoplasm comprised cuboidal to columnar to polygonal epithelial cells that form irregular tubules and tubulopapillary arrangements supported by moderate to abundant collagenous stroma (H&E, × 0.5, inset, H&E: × 40). (C) It consisted of a dense cellular neoplasm with vague streams of oval to spindle cells surrounded by small amounts of unmineralized and mineralized chondroid or mixoid matrix. The neoplastic cells have variably distinct cellular borders, moderate eosinophilic, fibrillar, or vacuolated cytoplas m, and round to oval nuclei with finely stippled chromatin and 1–2, nucleoli. There is mild to moderate anisocytosis (H&E, × 2, inset: H&E, × 40). (D) There was dense cellular and infiltrative proliferation of neoplastic round-to-spindle cells arranged in sheets within a variably dense fibrovascular stroma, leading to the subcutaneous mass expansion. Neoplastic cells vary from round to polygonal to spindle, have variably distinct cell borders, and contain moderate amphophilic to stippled basophilic cytoplasm. Anisokaryosis and anisokaryosis are marked (H&E, × 0.5, inset: H&E, × 40).
(TIF)

S2 Fig Computed tomography images of the tumors in four dogs. (A) Coronal and (B) Sagittal post-contrast image of dog 1 with the urethral carcinoma. The CT scan revealed an irregularly shaped and marginated mass (arrow) protruding into the lumen of the urinary bladder at the trigone level, involving the proximal urethra. (C) Axial and (D) Sagittal post-contrast image of dog 2 with intestinal adenocarcinoma. The CT scan demonstrated circumferential thickening of the proximal jejunum wall (arrow), characterized by heterogeneous contrast enhancement (pre 47HU, arterial 115HU, portal 123HU, delay 125HU). An enlargement of the adjacent jejunal lymph nodes (arrowhead) was also noted. (E) Coronal and (F) Sagittal post-contrast image of dog 3 with nasal chondrosarcoma. A well-defined, oval-shaped, isoattenuating destructive mass at the left caudal nasal cavity level was observed (arrow). Notably, the mass extended towards the left orbit on the left side, the right nasal cavity on the right side, the cranial cavity and nasopharynx on the caudal aspect, and the oral cavity on the ventral aspect. (G) Coronal post-contrast and (H) Sagittal pre-contrast image of dog 4 with rhabdomyosarcoma. The CT scan demonstrated a faint contrast-enhancing (pre 25HU, post 33HU), slightly inhomogeneous soft-tissue attenuating mass extending from the level of the head of the fibula to the distal 1/5 of the tibia on the lateral aspect of the right fibula (arrow). Aggressive mixed periosteal production and osteolysis of the adjacent right fibula were identified (arrowhead).
(TIF)

S1 Table Catalog of the 52 canids genomes. (XLSX)

S2 Table Detected SNVs and their genomic location in four solid tumors. (XLSX)

S3A Table The genomic locations and predicted impacts of SNVs with moderate to high impact in the carcinoma group. (XLSX)

S3B Table The genomic locations and predicted impacts of SNVs with moderate-to-high-impact in the sarcoma group. (XLSX)

S4 Table Detected SNVs and their genomic location in oncogenes from four solid tumors. (XLSX)

S5A Table The public data information. (XLSX)

S5B Table Our SNVs in public data. (XLSX)
