# Supplementary figures and images for: Identifying functional roles and pathways of shared mutations in canine solid tumors by whole-genome sequencing
Source: PLoS One. 2025 May 30;20(5):e0307792. doi: 10.1371/journal.pone.0307792 (PMC12124556; doi:10.1371/journal.pone.0307792)

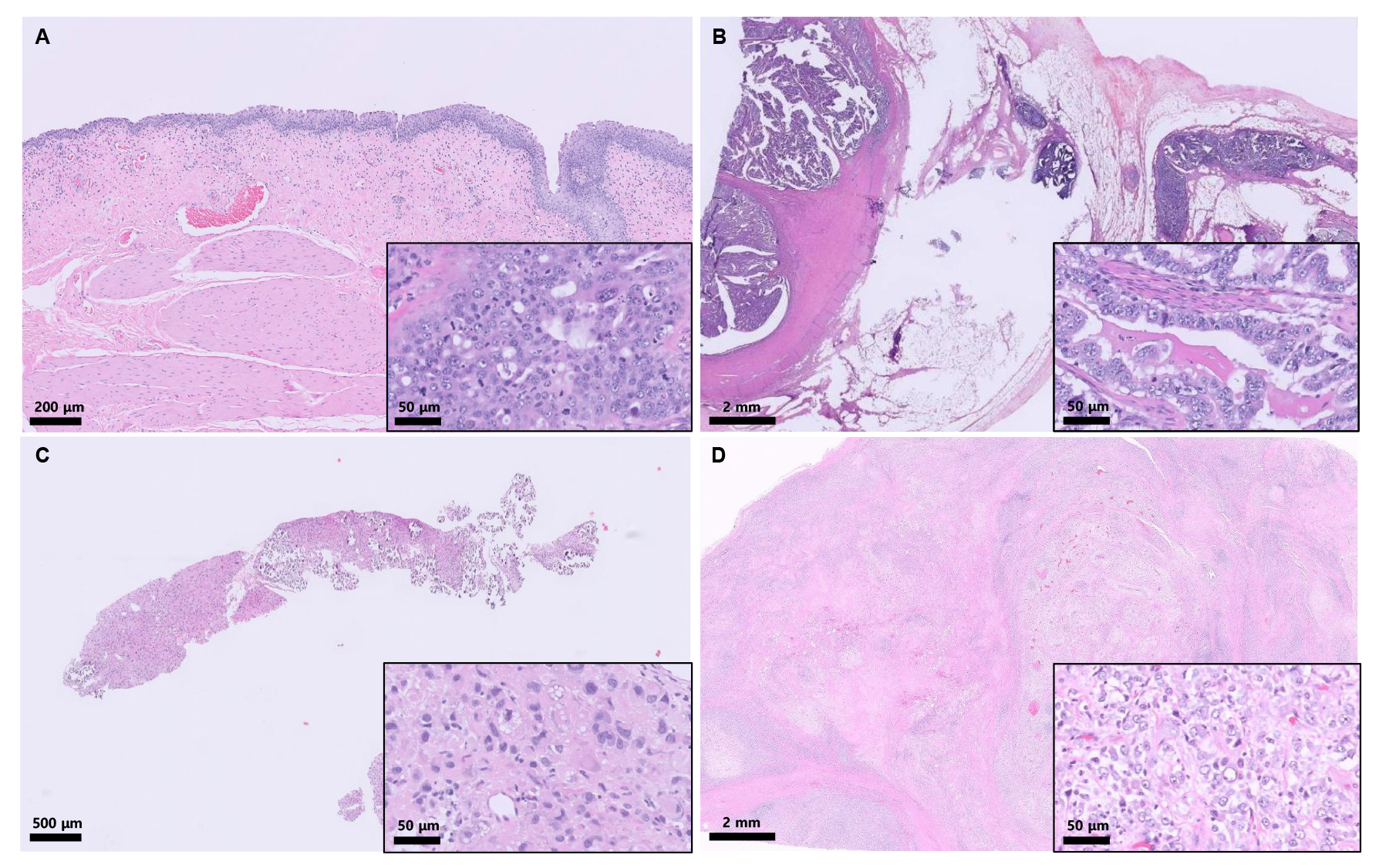

Supplement: S1 Fig — (A) Histopathological observation of the bladder mass (dog1), (B) intestinal mass (dog 2), nasal mass (dog 3), and the mass from the right hindlimb (dog 4). (A) It revealed highly invasive tumor that extended transmurally throughout bladder wall and disrupted the normal architecture. Neoplastic epithelial cells were polygonal with distinct cell borders and abundant eosinophilic cytoplasm. There is marked anisocytosis and anisokaryosis (H&E, × 5, inset: H&E, × 40). (B) Intestinal mucosa was regionally infiltrated and expanded by a poorly demarcated and markedly infiltrative neoplastic mass extending through intestinal wall segments. The neoplasm comprised cuboidal to columnar to polygonal epithelial cells that form irregular tubules and tubulopapillary arrangements supported by moderate to abundant collagenous stroma (H&E, × 0.5, inset, H&E: × 40). (C) It consisted of a dense cellular neoplasm with vague streams of oval to spindle cells surrounded by small amounts of unmineralized and mineralized chondroid or mixoid matrix. The neoplastic cells have variably distinct cellular borders, moderate eosinophilic, fibrillar, or vacuolated cytoplas m, and round to oval nuclei with finely stippled chromatin and 1–2, nucleoli. There is mild to moderate anisocytosis (H&E, × 2, inset: H&E, × 40). (D) There was dense cellular and infiltrative proliferation of neoplastic round-to-spindle cells arranged in sheets within a variably dense fibrovascular stroma, leading to the subcutaneous mass expansion. Neoplastic cells vary from round to polygonal to spindle, have variably distinct cell borders, and contain moderate amphophilic to stippled basophilic cytoplasm. Anisokaryosis and anisokaryosis are marked (H&E, × 0.5, inset: H&E, × 40). (TIF) [file pone.0307792.s001.tif]

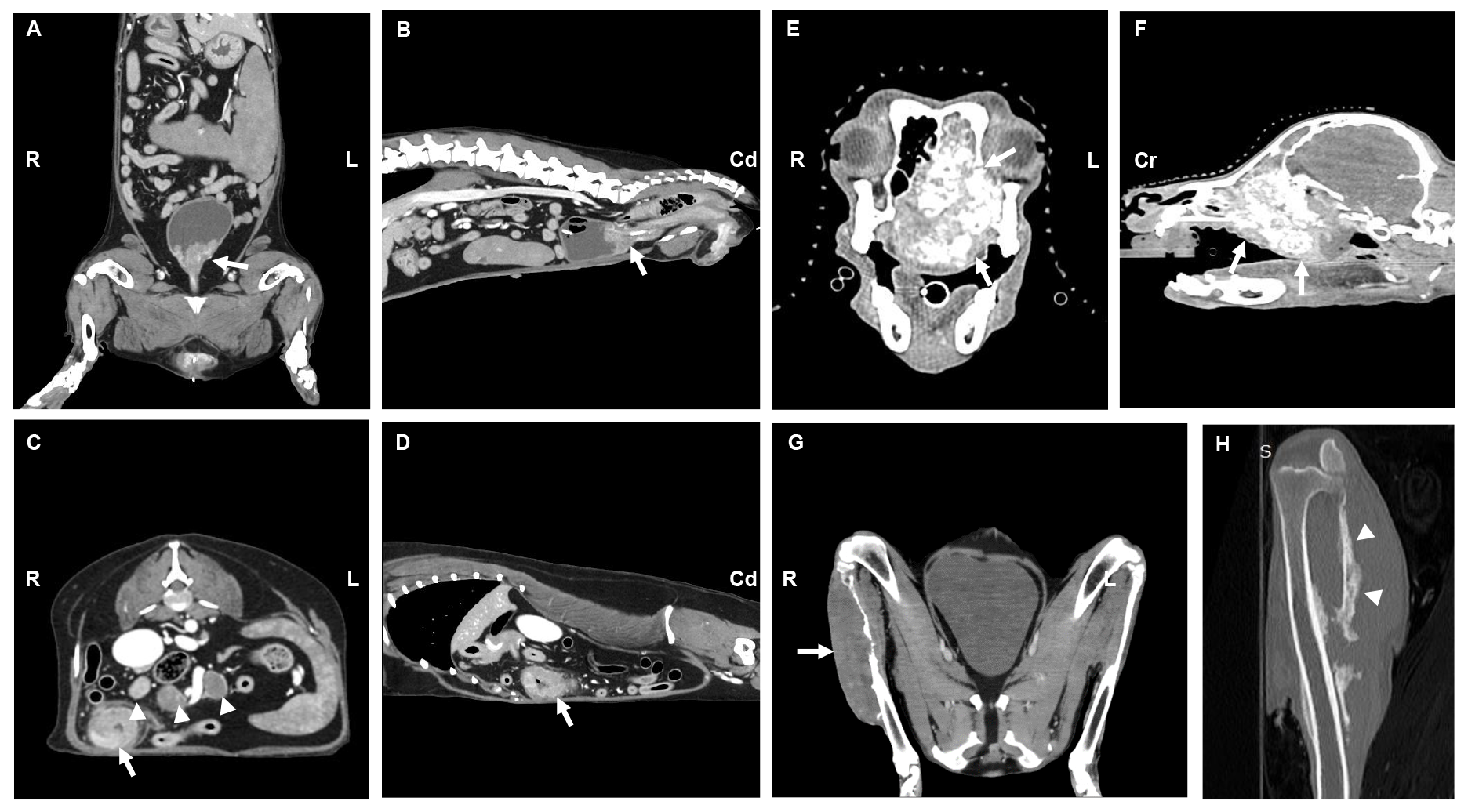

Supplement: S2 Fig — (A) Coronal and (B) Sagittal post-contrast image of dog 1 with the urethral carcinoma. The CT scan revealed an irregularly shaped and marginated mass (arrow) protruding into the lumen of the urinary bladder at the trigone level, involving the proximal urethra. (C) Axial and (D) Sagittal post-contrast image of dog 2 with intestinal adenocarcinoma. The CT scan demonstrated circumferential thickening of the proximal jejunum wall (arrow), characterized by heterogeneous contrast enhancement (pre 47HU, arterial 115HU, portal 123HU, delay 125HU). An enlargement of the adjacent jejunal lymph nodes (arrowhead) was also noted. (E) Coronal and (F) Sagittal post-contrast image of dog 3 with nasal chondrosarcoma. A well-defined, oval-shaped, isoattenuating destructive mass at the left caudal nasal cavity level was observed (arrow). Notably, the mass extended towards the left orbit on the left side, the right nasal cavity on the right side, the cranial cavity and nasopharynx on the caudal aspect, and the oral cavity on the ventral aspect. (G) Coronal post-contrast and (H) Sagittal pre-contrast image of dog 4 with rhabdomyosarcoma. The CT scan demonstrated a faint contrast-enhancing (pre 25HU, post 33HU), slightly inhomogeneous soft-tissue attenuating mass extending from the level of the head of the fibula to the distal 1/5 of the tibia on the lateral aspect of the right fibula (arrow). Aggressive mixed periosteal production and osteolysis of the adjacent right fibula were identified (arrowhead). (TIF) [file pone.0307792.s002.tif]
