# Supplementary material for: Identifying functional roles and pathways of shared mutations in canine solid tumors by whole-genome sequencing
Source: PLoS One. 2025 May 30;20(5):e0307792. doi: 10.1371/journal.pone.0307792 (PMC12124556; doi:10.1371/journal.pone.0307792)
Supplement: Attachment — Submitted filename: response_to_reviewers.docx [file pone.0307792.s011.docx]

Dear Editor,

Thank you for the opportunity to revise our paper by Jeon et al. entitled “Identifying Functional Roles and Pathways of Shared Mutations in Canine Solid Tumors by Whole-Genome Sequencing.” We specifically thank the reviewers for their careful review of our previously submitted version of this paper. Our paper is undoubtedly strengthened by the insightful comments. We have responded to all comments as follows.

**Comment #1 of Reviewer 1**

The study could be better if its experimental design could be further considered. Firstly, the sample size is limited, with only four dogs representing two distinct tumor groups (carcinomas and sarcomas). This small sample size restricts the generalizability of the findings and may need to capture the genetic diversity of canine tumors adequately. Additionally, the study lacks a detailed explanation of potential biases introduced by random selection or variation in tumor progression among the dogs. The absence of functional validation for the identified mutations, such as in vitro or in vivo experiments to confirm their roles in tumor development, limits the study’s ability to establish direct causal links between mutations and tumor pathways. Here are several potential research questions that could arise from this study:

**Response to Comment #1**

We agree that the small sample size in our study limits the generalizability of the findings. The inclusion of only four dogs was primarily constrained by the availability of suitable tumor samples with corresponding high-quality whole-genome sequencing data. Despite this limitation, our study provides an initial exploration into the genetic landscape of canine solid tumors, identifying potential trends and pathways that warrant further investigation.

To further expand our findings, we utilized the whole genome sequences of the two osteosarcoma samples (PRJNA525883) and three urothelial carcinoma samples (PRJNA1007700) publicly available on NCBI. We then aimed to investigate the presence of tumor-specific mutations identified in the current study on five additional tumor genomes.

|  | | | | **Urothelial carcinoma** | | | **Osteosarcoma** | |
| --- | --- | --- | --- | --- | --- | --- | --- | --- |
| **CHR** | **POS** | **REF** | **ALT** | **SAMN37094924** | **SAMN37094923** | **SAMN37094922** | **SAMN11079524** | **SAMN11079500** |
| 3 | 92482211 | A | C | 0/0 | 0/0 | 0/0 | 0/0 | 0/1 |
| 3 | 92482217 | A | C | 0/0 | 0/0 | 0/0 | 0/0 | 0/1 |
| 6 | 36875327 | C | A | 0/0 | 0/1 | 0/1 | 0/0 | 0/0 |
| 6 | 36875345 | C | G | 0/0 | 0/1 | 0/1 | 0/0 | 0/0 |
| 6 | 36875456 | T | C | 0/0 | 0/1 | 0/1 | 0/0 | 0/0 |
| 20 | 45239123 | C | T | 0/0 | 1/1 | 0/0 | 0/1 | 1/1 |
| 20 | 46629545 | A | C | 0/0 | 0/1 | 0/0 | 0/0 | 0/0 |

Our goal was to examine the presence of tumor-specific mutations identified in our study across five additional tumor genomes. We discovered that, of the 96 candidate variants, 5 single nucleotide variants (SNVs) (3:92482211, 3:92482217, 6:36875327, 6:36875345, 6:36875456) found in carcinoma and 2 SNVs (20:45239123, 20:46629545) found in sarcoma were also present in at least one tumor genome. While the overlap between different cancer types was modest, these seven variants may support the validity of our approach and suggest a potential genetic predisposition to a wide range of tumor types. We have discussed the corresponding results on pages 6 and 15.

**Comment #2 of Reviewer 1**

There are some questions the reviewer wants to clarify further and might need the authors to carefully validate or discuss them.

1. In addition to CanFam 1.0, other canine genome sequencing references include CanFam 2.0, CanFam 3.1, the Dog10K project, and breed-specific references like Shib 2.0 for Shiba Inu. These updated versions offer improved accuracy, broader genetic data, and tools for studying breed-specific traits and diseases, providing valuable resources for canine genetic research. So, what are the potential limitations of using only a single canine genome reference, such as CanFam 1.0, for analysis in this study, particularly regarding genetic diversity, breed-specific mutations, and the accuracy of variant detection across different tumor types?

**Response to Comment #2**

Thank you for highlighting the importance of the reference genome choice in our study. In this paper, we utilized ROS_Cfam_1.0, a recently developed high-quality reference genome (released on Sep 3, 2020). A single reference genome cannot fully capture all the variations present across diverse breeds and tumors, which may lead to some variations being miscalled or missed. newer reference genomes, such as CanFam4 (UU_Cfam_GSD_1.0), CanFam5 (UMICH_Zoey_3.1), and CanFam6 (Dog10K_Boxer_Tasha), also exist. According to previously published studies, when the entire CanFam3.1 genome was converted to align with more recent reference genomes, such asCanFam4 (UU_Cfam_GSD_1.0), CanFam5 (UMICH_Zoey_3.1), and CanFam6 (Dog10K_Boxer_Tasha), high mapping feasibility and low lift-over failure rates were observed. This indicates the high quality and similarity between reference genomes, suggesting that when results are generated using a single reference genome, similar outcomes are likely to be obtained with other reference genomes [1]. We discussed this issue in the revised manuscript on pages 36-37.

**Comment #3 of Reviewer 1**

2. What are the specific roles of immune-related mutations in the development and metastasis of canine solid tumors, and can these pathways be targeted therapeutically?

**Response to Comment #3**

Tumors are generally considered to be deeply connected with the immune system. Indeed, many tumors are known to have the ability to evade immunity or create an immunosuppressive tumor microenvironment. Through these mechanisms, tumors can develop and metastasize. Additionally, chronic inflammation can serve as a cause of cancer development.

A notable example is mutations in the TP53 gene, which can enable tumor cells to develop immune evasion mechanisms. According to previously published studies, p53-mutated cells exhibit evasion from NK cells through the reduced expression of NK-activating ligands and suppression of the cGAS-STING pathway [2]. Additionally, p53-mutated cells contribute to immune evasion by releasing extracellular vesicles expressing PD-L1, which suppress macrophage phagocytosis. These mutated cells also promote tumor growth by recruiting Tregs (regulatory T cells) to the tumor microenvironment (TME), which inhibits anti-tumor cells such as cytotoxic CD8+ T cells. The TME plays a critical role in tumor metastasis and significantly contributes to immune evasion [3].

Identifying such immune-related mutations and the immune pathways affected by these mutations can enable the development of targeted therapies. In some canine solid tumors, high PD-L1 expression has been observed, suggesting the potential for developing new therapies targeting the PD-L1 pathway [4].

In this study, mutations were identified in NLRP12, TNIP2, and IRAK4, genes associated with the NF-κB signaling pathway. According to previous studies, the NF-κB signaling pathway directly contributes to the development of various solid tumors and plays an essential role in inflammation and innate immunity [5]. Efforts are currently underway to develop NF-κB inhibitors for cancer therapy [6]. Therefore, targeting pathways associated with immune-related mutations as therapeutic targets is considered a highly valid approach. We discussed this in the revised manuscript on page 26.

**Comment #4 of Reviewer 1**

Can the identified genetic mutations in canine tumors be directly linked to similar mutations in human cancers, providing potential translational insights for both veterinary and human oncology?

**Response to Comment**

Dogs and humans are not only exposed to similar environments but also share similarities in tumor development. For example, mutations in genes identified in canine mammary cancer are also observed in human breast cancer. A notable example is the p.H1047R hotspot mutation in the *PIK3CA* gene, which is well-known in human breast cancer and has also been found in canine mammary cancer [7]. In addition, common mutations have been observed in both canine and human tumors in genes such as *KRAS*, *NRAS*, *BRAF*, *KIT*, and *EGFR* [8]. The presence of these shared mutations suggests that the process of cancer development may be similar in dogs and humans, making canine models valuable for studying cancer and developing treatments. Furthermore, for cancers that are rare or difficult to sample in humans, similar canine tumors can be utilized for research, providing valuable insights into these types of cancers. We have discussed this in the revised manuscript on page 35.

**Comment #5 of Reviewer 1**

4. Would larger-scale studies with more diverse tumor types confirm the findings, or would additional genetic variations and pathways be discovered in a broader canine population?

**Response to Comment**

It is highly probable that further genetic variations and pathways could be uncovered. Analyzing a broader array of tumor types would also enable us to validate the mutations or genes identified in our study across other tumors (response to comment #1). The discovery of common mutations in different tumors may indirectly suggest the existence of shared mechanisms despite differences in tumor types. By utilizing a larger and more diverse canine population, the impact of breed-specific effects could be further minimized. Moreover, if the mutations we identified are also found in other breeds, it would provide more robust evidence to generalize our findings.

**References**

1.	Son KH, Aldonza MBD, Nam AR, Lee K-H, Lee J-W, Shin K-J, et al. Integrative mapping of the dog epigenome: Reference annotation for comparative intertissue and cross-species studies. Science Advances. 2023;9(27). doi: 10.1126/sciadv.ade3399.

2.	Wang C, Tan JYM, Chitkara N, Bhatt S. TP53 Mutation-Mediated Immune Evasion in Cancer: Mechanisms and Therapeutic Implications. Cancers. 2024;16(17):3069. doi: 10.3390/cancers16173069.

3.	Biray Avci C, Goker Bagca B, Nikanfar M, Takanlou LS, Takanlou MS, Nourazarian A. Tumor microenvironment and cancer metastasis: molecular mechanisms and therapeutic implications. Frontiers in Pharmacology. 2024;15. doi: 10.3389/fphar.2024.1442888.

4.	Maekawa N, Konnai S, Ikebuchi R, Okagawa T, Adachi M, Takagi S, et al. Expression of PD-L1 on Canine Tumor Cells and Enhancement of IFN-γ Production from Tumor-Infiltrating Cells by PD-L1 Blockade. PLoS ONE. 2014;9(6):e98415. doi: 10.1371/journal.pone.0098415.

5.	Hoesel B, Schmid JA. The complexity of NF-κB signaling in inflammation and cancer. Molecular Cancer. 2013;12(1):86. doi: 10.1186/1476-4598-12-86.

6.	Xia L, Tan S, Zhou Y, Lin J, Wang H, Oyang L, et al. Role of the NFκB-signaling pathway in cancer. OncoTargets and Therapy. 2018;Volume 11:2063-73. doi: 10.2147/ott.s161109.

7.	Arendt ML, Sakthikumar S, Melin M, Elvers I, Rivera P, Larsen M, et al. PIK3CA is recurrently mutated in canine mammary tumors, similarly to in human mammary neoplasia. Scientific Reports. 2023;13(1). doi: 10.1038/s41598-023-27664-7.

8.	Rodrigues L, Watson J, Feng Y, Lewis B, Harvey G, Post G, et al. Shared hotspot mutations in oncogenes position dogs as an unparalleled comparative model for precision therapeutics. Scientific Reports. 2023;13(1). doi: 10.1038/s41598-023-37505-2.
